# Acute Rheumatism

**Published:** 1900-02

**Authors:** 


### ACUTE RHEUMATISM.

Meier, G. C. H., (*American Therapist,*) states that his experience with the salicylates during the past twenty years has convinced him of the difficulty of administering them in many cases, even if given in capsules or wafers; many patients object to salicylate of sodium on account of the resulting disturbances of the stomach, as evidenced by nausea, distress in the epigastrium, and even vomiting. Occasionally, he has also witnessed headache, tinnitus and vertigo during its use. These disadvantages of the salicylates have led him to experiment with aspirin, a new combination of salicylate acid and acetyl, which passes unchanged through the stomach, and is not decomposed until it reaches the intestinal canal, thus avoiding any action upon the stomach. He has given aspirin in the same dose as salicylate of sodium, and owing to its insolubility has generally placed the desired dose upon a tablespoon, adding some sugar and water. From his experience with aspirin up to date, he considers himself able to assert that its effect differs in no wise from that of salicylate of sodium, and that it has the great advantage of being entirely devoid of troublesome effects upon the stomach. This is well illustrated by the twelve cases which are reported in full in his contribution.

